# Description of a new species of Eucinetidae (Coleoptera, Scirtoidea) from Cretaceous Burmese amber

**DOI:** 10.3897/zookeys.982.39335

**Published:** 2020-11-02

**Authors:** Xueyong Du, Adam Slipinski, Zhenhua Liu, Hong Pang

**Affiliations:** 1 State Key Laboratory of Biocontrol, School of Life Sciences, Sun Yat-sen University, Guangzhou 510275, China; 2 State Key Laboratory of Biocontrol, School of Ecology, Sun Yat-sen University, Guangzhou 510275, China; 3 Australian National Insect Collection, CSIRO, GPO Box 1700, Canberra, ACT 2601, Australia

**Keywords:** *
Eucinetus
*, generic diagnosis, relationship

## Abstract

*Eucinetus parvus***sp. nov.** is described from late Cretaceous Burmese amber, representing the second record of Eucinetidae from the Burmese amber and the first species of the family with simple, not piercing, mouthparts. A comparison between Mesocinetidae and Eucinetidae is provided.

## Introduction

The family Eucinetidae Lacordaire, 1857 is a relatively small group in the polyphagan Coleoptera, consisting of 10 extant genera and fewer than 60 species distributed worldwide (Leschen 2016; Lawrence 2019). The genera of living Eucinetidae, *Nycteus* Latreille, 1829, *Bisaya* Reitter, 1884, *Euscaphurus* Casey, 1885, *Jentozkus* Vít, 1977, *Tohlezkus* Vít, 1977, *Eucilodes* Vít, 1985, *Eucinetella* Nikitsky, 1996, *Proeuzkus* Vít, 2000, and *Noteucinetus* Bullians & Leschen, 2004, with exception of *Eucinetus* Germar, 1818, are mostly very limited in their geographic distribution and contain only a few species. Because of the peculiar shape of the metacoxae, these beetles are usually called “plate-thigh beetles” (Guéorguiev 2004). The living species can usually be found under bark or are extracted from leaf litter, and they have been found in a relationship with myxomycete or basidiomycete fungi on which adult beetles and larvae probably feed (Leschen 2016). Historically, Eucinetidae has been included in the superfamily Dascilloidea (Crowson 1955), but Crowson (1960) created Eucinetoidea for Eucinetidae, Scirtidae, and Clambidae based on both adult and larval characters; Lawrence and Newton (1982) accepted this systematic arrangement, while Lawrence and Newton (1995) later claimed the priority of Scirtoidea, which also included Decliniidae Nikitsky, Lawrence, Kirejtshuk & Gratshev, 1994 (Nikitsky et al. 1994; Lawrence et al. 1995). Molecular studies (McKenna et al. 2015; Zhang et al. 2018) also supported the close relationships of these families, together with Derodontidae, and they indicated that Eucinetidae may be close to the basal group in Polyphaga. Within the family, species of *Bisaya*, *Jentozkus*, *Tohlezkus*, *Eucilodes*, and *Proeuzkus* have subsuctorial mouthparts, the function of which remains unclear until now.

The fossil record of Eucinetidae is sparse. The fossil genus *Mesocinetus* Ponomarenko, 1986, which was described from the early Cretaceous of the Gurban-Eren Formation in western Mongolia (Ponomarenko 1986), was subsequently placed in its own family, Mesocinetidae (Kirejtshuk and Ponomarenko 2010). The family Mesocinetidae was thought to be related to Scirtidae and Eucinetidae, and included four other late-Jurassic genera (*Manoelodes* Kirejtshuk & Ponomarenko, 2010, *Manopsis* Kirejtshuk & Ponomarenko, 2010, *Parashartegus* Kirejtshuk & Ponomarenko, 2010, and *Shartegus* Kirejtshuk & Ponomarenko, 2010). Two species of the genus *Huaxiacinectus* Hong, 1995, which were described from early Cretaceous of the Huachi-Huanhe Formation in China (Hong 1995), were attributed to Eucinetidae, although the true attributions of these species remain unknown and need study. Jałoszynski (2019) recently described a eucinetid specimen from Burmese amber with highly modified piercing mouthparts as *Cretohlezkus alleni* Jałoszynski, 2019. The oldest species of the genus *Eucinetus* was in Bembridge Marls from the late Eocene of the Isle of Wight (Kirejtshuk et al. 2019).

Here, a new fossil species of the extant genus *Eucinetus* (Eucinetidae) with simple mouthparts from Burmese amber is presented. This new species demonstrates the ancient origin of this lineage of beetles.

## Materials and methods

The specimen included in this study is embedded in Burmese amber from the Hukawng Valley of northern Myanmar (Cruickshank and Ko 2003; Dong et al. 2015: fig. 1). The age of this amber is generally considered to be near the Albian/Cenomanian (98.79 ± 0.62 Ma) (Shi et al. 2012). The amber specimen is deposited in the Museum of Biology, Sun Yat-sen University, China (**SYSBM**).

For preparation, the amber material was polished with emery papers of various grits and polished with polishing powder. Images were taken using a Nikon DS-Ri2 camera mounted on a Nikon SMZ25 microscope; layers were captured and aligned using NIS-Elements software and processed in Photoshop CC. The line drawings were prepared in Adobe Illustrator CC and figures were compiled in Photoshop CC. The length of the beetle specimen was measured from the anterior margin of head to the apex of elytra; the width is the maximum width of the elytra. Morphological terminology of Eucinetidae follow Leschen (2016).

## Systematic paleontology

### Superfamily Scirtoidea Fleming, 1821

**Family Eucinetidae Lacordaire, 1857**

#### Eucinetus

Taxon classificationAnimaliaColeopteraEucinetidae

Germar, 1818

A6A5A8A1-2729-51B0-8606-C22AAEEF94A6

##### Type species.

*Scaphidium haemorrhoidalis* Germer, 1818.

##### Diagnosis.

*Eucinetus* can be separated from *Bisaya*, *Eucilodes*, *Jentozkus*, *Proeuzkus*, *Tohlezkus*, and *Cretohlezkus* by having the simple labium. Among the other genera, it differs from *Eucinetella* and *Euscaphurus* in the filiform antenna and broad labrum, and from *Noteucinetus* in the slender body shape and transverse rows of striae on elytra. *Eucinetus* is most similar to *Nycteus* except that antennomere 3 in *Nycteus* is distinctly shorter than the adjacent segments, which is almost the same length as antennomere 4 in *Eucinetus*.

#### Eucinetus parvus sp. nov.

Taxon classificationAnimaliaColeopteraEucinetidae

DA8B65B5-4A57-5222-A83C-A2E940C695ED

http://zoobank.org/2E2CC58D-59B7-4AB1-AB3F-64DD3D733225

Figures 1 , 2

##### Etymology.

Latin, *parvus*, meaning small, which refers to the small body size of the new species.

##### Holotype.

SYS-ENAM0011, female.

##### Locality and horizon.

Hukawng Valley, Kachin State, northern Myanmar; lowermost Cenomanian, Upper Cretaceous.

##### Diagnosis.

The new species can be distinguished from all the extant species of *Eucinetus* by the combination of the following characters: relatively small and narrower body; slenderer mesepimeron and matanepisternum; relatively short antenna with the scape shorter than pedicel. It can also be easily separated from *Eucinetus nikolaevae* by much smaller body (1.9 mm long compared to 2.8 mm in *E. nikolaevae*), slender body-shape, and sub-rectangular labrum.

##### Description.

**Figure 1.**
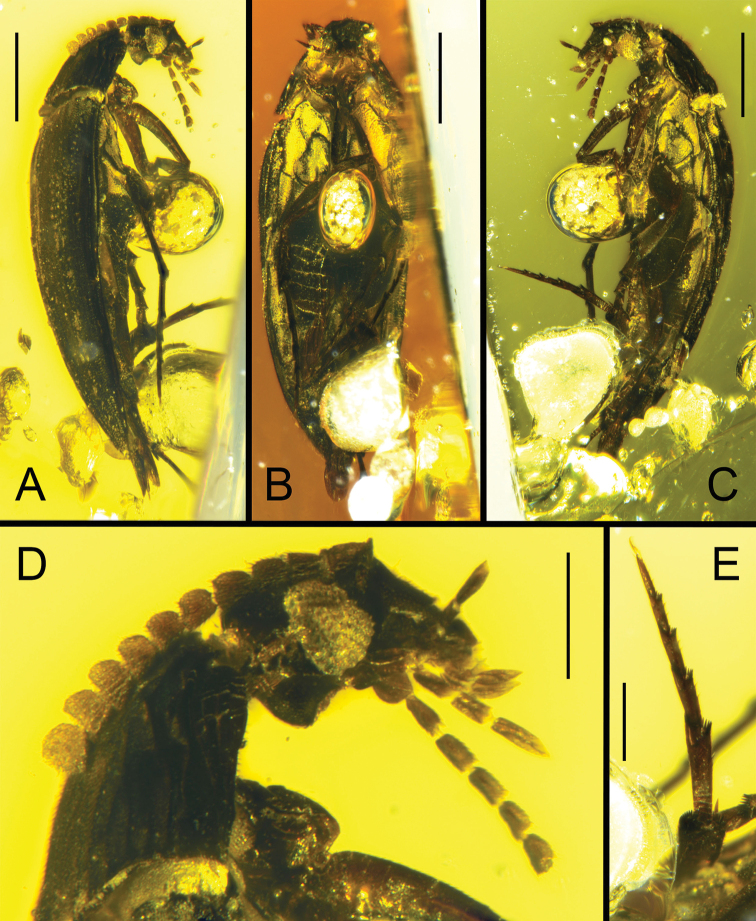
*Eucinetus parvus* sp. nov., holotype SYS-ENAM0011 **A, C** lateral view, opposite **B** ventral view **D** head, antennae, maxillary and labial palps **E** hind tarsi. Scale bars: 0.5 mm (**A–C**); 0.2 mm (**D, E**).

Length about 1.9 mm, width 0.7 mm. Body elongate-fusiform (Fig. 1A–C), black, dorsum slightly convex, and covered with dense, short setae.

Head relatively small, subtriangular; posterior margin nearly truncate. Eyes moderately large, protruding, and finely facetted. Temple behind eyes moderately long; posterior angles orthogonal. Antennae closely inserted in front of eyes in antennal fossae. Antenna (Fig. 1D) 11-segmented, short, and not extending beyond posterior margin of pronotum; scape relatively small and longer than wide; pedicel dilated and distinctly wider than adjacent segments; antennomere 3 nearly as long as following antennomeres; antennomeres 3–10 increasingly transverse toward apex; terminal antennomere larger with rounded apical margin. Frontoclypeal suture absent; labrum long, wide, and subrectangular, with nearly straight apical margin. Maxillary palp (Fig. 1D) 4-segmented; first segment very short; palpomeres 2 and 3 subequal and cylindrical; terminal segment longest, not wider than penultimate one, and fusiform anteriorly, with sharp apex. Labium with simple prementum; labial palp 3-segmented; terminal palpomere largest and fusiform, with apex elongate and sharp.

Pronotum transverse, widest posteriorly; anterior margin broadly rounded, lateral margins gradually widened posteriorly, and posterior margins sinuate; disc with distinct microsculpture, uniformly covered by dense, short setae. Prosternum highly reduced with very narrow area in front of procoxae; prosternal process narrow. Notosternal suture present. Procoxae strongly transverse and projecting, nearly contiguous, and protrochantins exposed; procoxal cavities externally widely opened.

**Figure 2.**
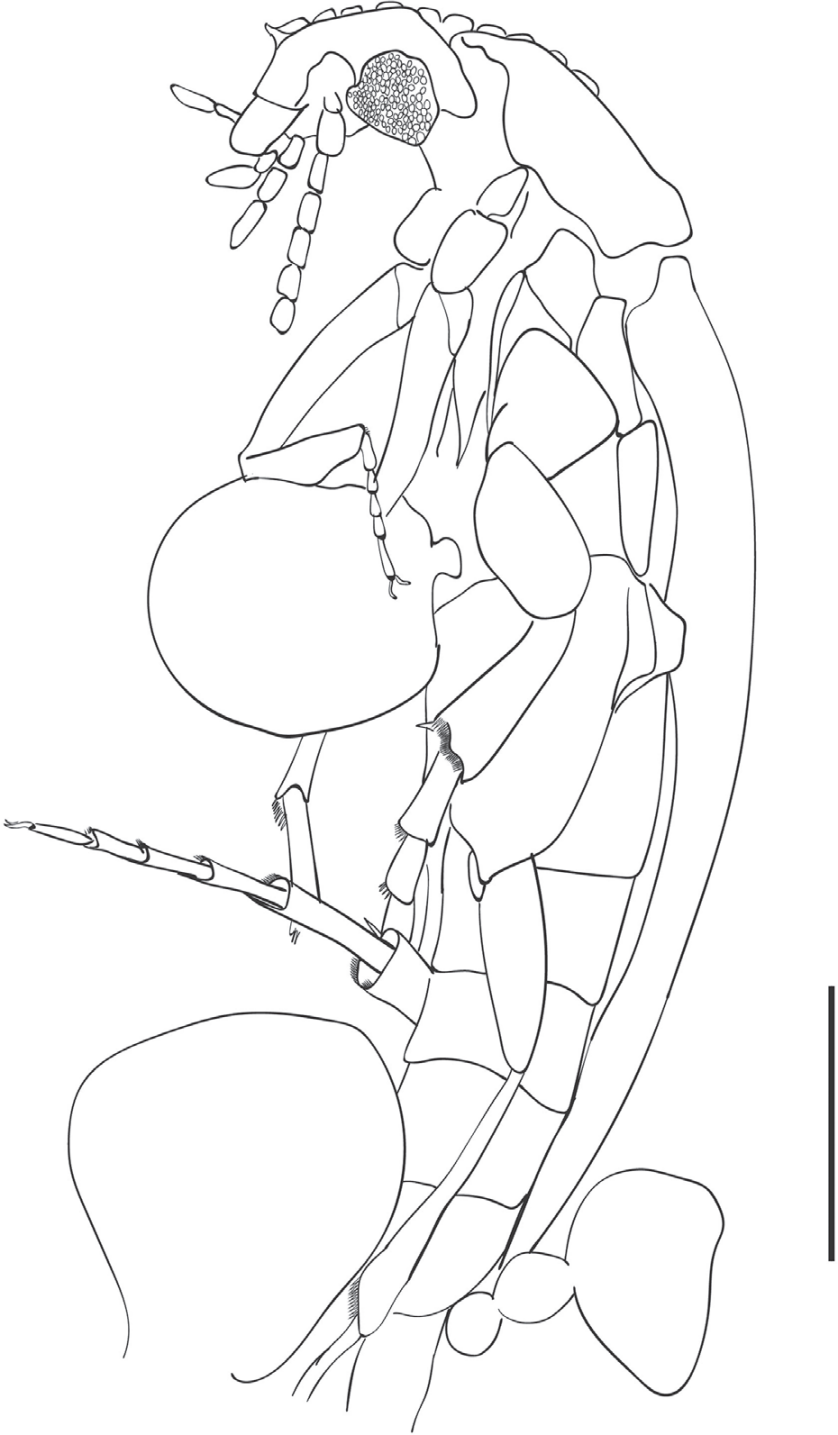
*Eucinetus parvus* sp. nov., holotype SYS-ENAM0011, line drawing. Scale bar: 0.5 mm.

Elytra elongate, about 2.3 times as long as wide, lateral margins gradually narrowed posteriorly, apex sharp; dorsal surface with dense, distinct, transverse microsculpture and covered by dense, uniform setae; epipleuron not extending to apex and relatively narrow at base. Mesoventrite short, with deep, longitudinal, middle depression fitting fore femur. Mesanepisternum large and subrectangular; mesepimeron large and subtrapezoid. Mesocoxal cavities (Figs 1C, 2) large and subovate; laterally widely open to mesepimeron; mesocoxae moderately separated and not projecting; mesotrochantins concealed. Metaventrite short, transverse, and not narrowed towards lateral margins; metanepisternum subtriangular and elongated. Metacoxae contiguous, with large metacoxal plates covering hind femora and most of abdominal ventrite 1; metacoxal plates with anterior margin only slightly oblique, lateral margins curved. Tarsal formula 5-5-5. Abdomen with five visible ventrites, terminal ventrite subtriangular. Fore leg with small, indistinct trochanter; femur elongate and slightly curved; tibia short and flattened, gradually widened toward apex; apical spurs highly reduced; tarsi 5-segmented, with basal four tarsomeres short and almost in same length; claws small. Mid leg with small trochanter and enlarged femur; tibia flattened and broadened apically, with pair of apical spurs unequal in length; apex with fringe of small spines; tarsus with first tarsomere longest, nearly the same length as following two segments combined, tarsomeres 2–5 gradually shortened, tarsomeres 2–4 with fringe of spines apically. Hind leg (Fig. 1E) with femur elongate and slightly dilated, mostly concealed by metacoxal plate; tibia longer than femur, flattened and widened apically, and apex with pair of short unequal apical spurs and fringe of spines; first tarsomere longest and almost same length as following two segments combined; tarsomeres 2–4 gradually shortened; last tarsomere with pair of small claws, nearly same length as penultimate one.

## Discussion

The placement of the new species in Eucinetidae is based on the combination of hypognathous head, fusiform body shape, 5-5-5 tarsal formula, fringes of spines on apex of tibiae and tarsomeres, and huge metacoxal plates. Unlike *Cretohlezkus*, which was described from Burmese amber (Jałoszynski 2019), the new species has a simple prementum and a relatively long metaventrite and metepimeron, which are similar to those in some species of *Eucinetus* and *Nycteus*. Antennomere 3 in *Nycteus* is distinctly shorter than the following segment, different than in the new species. The characters discussed above suggest the placement of the new fossil taxon in the genus *Eucinetus*. However, the diagnosis of this genus is very broad and requires further research. The occurrence of Eucinetidae in Burmese amber with both simple and piercing mouthparts suggests that the family habits were already very diverse 99 Ma ago and this diversity has been retained to the present.

Eucinetidae is closely related to the fossil family Mesocinetidae, which is distinguished mostly by the large metacoxal plates, very short metaventrite, and widened metanepisternum (Vít 1995: fig. 10, 1997: figs 20, 21, 2000: fig. 3). Mesocinetidae differs from Eucinetidae in having small metacoxal plates, a relatively long metaventrite, and a subrectangular metepimeron (Kirejtshuk and Ponomarenko 2010). However, the differences between these families need further research, as intermediate taxa are found in Eucinetidae. The extant genus *Eucilodes* is similar to Mesocinetidae in sharing some of those characters (Vít 1985: figs 5, 21), but it has piercing mouthparts absent from known taxa of Mesocinetidae. Similarly, some species of *Eucinetus* and *Nycteus* have relatively long metaventrites and metanepisterna (Vít 1977: figs 1, 3, 1979: figs 9, 10, 1985: fig. 7), while in other species of the same genera these structures are much shorter (Vít 1990: fig. 28). The genera of Eucinetidae with subsuctorial mouthparts was found to be monophyletic in Jałoszynski’s (2019) morphological phylogenetic analysis, but more comprehensive samples and molecular data are required to verify this hypothesis.

## Supplementary Material

XML Treatment for Eucinetus

XML Treatment for Eucinetus parvus

## References

[B1] Bullians MS Leschen RAB (2004) *Noteucinetus* new genus from New Zealand and Chile and notes on *Eucinetus stewarti* (Broun) (Coleoptera: Eucinetidae). New Zealand Entomologist 27: 29–38. 10.1080/00779962.2004.9722121

[B2] Casey TL (1885) New genera and species of Californian Coleoptera. Bulletin of the California Academy of Sciences 1: 283–336. 10.5962/bhl.title.8839

[B3] Crowson RA (1955) The Natural Classification of the Families of Coleoptera. Holywell Press, Oxford, 187 pp.

[B4] Crowson RA (1960) The phylogeny of Coleoptera. Annual Review of Entomology 5: 111–134. 10.1146/annurev.en.05.010160.000551

[B5] Cruickshank RD Ko K (2003) Geology of an amber locality in the Hukawng Valley, northern Myanmar. Journal of Asian Earth Sciences 21(5): 441–455. 10.1016/S1367-9120(02)00044-5

[B6] Dong F Shih C Ren D (2015) A new genus of Tanyderidae (Insecta: Diptera) from Myanmar amber, upper cretaceous. Cretaceous Research 54: 260–265. 10.1016/j.cretres.2014.12.011

[B7] Germar EF (1818) Vermischte Bemerkungen über einige Käferarten. Magazin der Entomologie 3: 228–260.

[B8] Guéorguiev B (2004) Eucinetidae – a new family to the fauna of Bulgaria (Coleoptera: Scirtoidea). Historia Naturalis Bulgarica 16: 113–117.

[B9] Hong YC (1995) Fossil insects of the southern ordos basin. Acta Geologica Gansu 4(1): 8–9. [in Chinese]

[B10] Jałoszynski P (2019) †*Cretohlezkus* gen. nov. from Upper Cretaceous Burmese amber demonstrates ancient origins of suctorial mouthparts in Eucinetidae (Coleoptera: Scirtoidea). Cretaceous Research 100: 126–133. 10.1016/j.cretres.2019.03.016

[B11] Kirejtshuk AG Ponomarenko AG (2010) A new coleopterous family Mesocinetidae fam. nov. (Coleoptera: Scirtoidea) from Late Mesozoic and notes on fossil remains from Shar-Teg (Upper Jurassic, South-Western Mongolia). Zoosystematica Rossica 19: 301–325.

[B12] Kirejtshuk AG Ponomarenko AG Kurochkin AS Alexeev AV Gratshev VG Solodovnikov AY Krell FT Soriano C (2019) The beetle (Coleoptera) fauna of the Insect Limestone (late Eocene), Isle of Wight, southern England. Earth and Environmental Science Transactions of the Royal Society of Edinburgh 110: 405–492. 10.1017/S1755691018000865

[B13] Latreille PA (1829) Crustacés, arachnides et partie des insectes. In: Cuvier G (Ed.) Le Règne Animal, Distribué D’après son Organisation, Pour Servir de Base a L’histoire Naturelle des Animaux et D’introduction à L’anatomie Comparée. Nouvelle édition, revue et augmentée. Tome IV. Deterville & Crochard, Paris, 584 pp.

[B14] Lacordaire JT (1857) Histoire Naturelle des Insectes. Genera des Coléopteres ou Exposé Méthodique et Critique de Tous les Genres Proposés Jusqu’ici Dans cet Ordre D’insectes. Tome quatrieme contenant les familles des buprestides, throscides, eucnémides, élatérides, cébrionides, cérophytides, rhipicérides, dascyllides, malacodermes, clérides, lyméxylones, cupésides, ptiniores, bostrichides et cissides. Librairie Encyclopédique de Roret, Paris, 579 pp.

[B15] Lawrence JF Newton AF (1982) Evolution and classification of beetles. Annual Review of Ecology and Systematics 13: 261–290. 10.1146/annurev.es.13.110182.001401

[B16] Lawrence JF Newton AF (1995) Families and subfamilies of Coleoptera (with selected genera, notes, references and data on family-group names). In: Pakaluk J Ślipiński SA (Eds) Biology, Phylogeny, and Classiﬁcation of Coleoptera: Papers Celebrating the 80th Birthday of Roy A.Crowson (Vol. 2). Muzeum i Instytut Zoologii Polska Akademia Nauk, Warsaw, 779–1006.

[B17] Lawrence JF Nikitsky NB Kirejtshuk AG (1995) Phylogenetic position of Decliniidae (Coleoptera: Scirtoidea) and comments on the classiﬁcation of Elateriformia (sensu lato). In: Pakaluk J Ślipiński SA (Eds) Biology, Phylogeny, and Classiﬁcation of Coleoptera: Papers Celebrating the 80th Birthday of Roy A.Crowson (Vol. 1). Muzeum i Instytut Zoologii Polska Akademia Nauk, Warsaw, 375–410.

[B18] Lawrence JF (2019) New species of *Eucinetus* and *Noteucinetus* from Australia (Coleoptera: Scirtoidea: Eucinetidae). Zootaxa 4668(2): 151–182. 10.11646/zootaxa.4668.2.1 31716625

[B19] Leschen RAB (2016) Eucinetidae Lacordaire, 1857. In: Beutel RG Leschen RAB (Eds) Handbook of Zoology, Vol.IV, Arthropoda: Insecta. Coleoptera, Vol. 1: Morphology and Systematics (Archostemata, Adephaga, Myxophaga, Polyphaga partim), Second Edition. Walter De Gruyter, Berlin/Boston, 206–210.

[B20] McKenna DD Wild AL Kanda K Bellamy CL Beutel RG Caterino MS Farnum CW Hawks DC Ivie MA Jameson ML Leschen RAB Marvaldi AE McHugh JV Newton AF Robertson JA Thayer MK Whiting MF Lawrence JF Ślipiński A Maddison DR Farrell BD (2015) The beetle tree of life reveals that Coleoptera survived end‐Permian mass extinction to diversify during the Cretaceous terrestrial revolution. Systematic Entomology 40(4): 835–880. 10.1111/syen.12132

[B21] Nikitsky NB (1996) New Coleoptera from China. Zoologicheskii Zhurnal 75: 1366–1373. [in Russian]

[B22] Nikitsky NB Lawrence JF Kirejtshuk AG Gratshev VG (1994) A new beetle family, Decliniidae fam. n., from Russian Far East and its taxonomic relationships (Coleoptera, Polyphaga). Russian Entomological Journal 2(5): 3–10.

[B23] Ponomarenko AG (1986) Insects in the Early Cretaceous ecosystems of the West Mongolia. Beetles – Scarabaeida (= Coleoptera). Trudy sovmestnoy sovetsko-mongol’skoy paleontologicheskoy ekspeditsii [Proceedings of the Joint Soviet-Mongolian Paleontological Expedition] 28: 84–105. [in Russian]

[B24] Reitter E (1884) Diagnosen neuer Coleopteren aus Lenkoran. Verhandlungen des Naturforschenden Vereins in Brün 22: 3–10.

[B25] Shi G Grimaldi DA Harlow GE Wang J Wang J Yang M Lei W Li Q Li X (2012) Age constraint on Burmese amber based on U-Pb dating of zircons. Cretaceous research 37: 155–163. 10.1016/j.cretres.2012.03.014

[B26] Vít S (1977) Contribution à la connaissance de la famille Eucinetidae (Coleoptera). Revue Suisse de Zoologie 84(4): 917–935. 10.5962/bhl.part.91368 897542

[B27] Vít S (1979) Deuxieme contribution a la connaissance du genre *Eucinetus* Germar (Col., Eucinetidae): revision des espèces de la région éthiopienne. Mitteilungen der Schweizerischen Entomologischen Gesellschaft [Bulletin de là Société Entomologique Suisse] 52: 409–415.

[B28] Vít S (1985) Étude de la morphologie des espèces paléarctiques du genre *Eucinetus* Germar et quelques remarques sur son utilisation taxonomique (ColeopteraEucinetidae). Annales de la Société suisse de Zoologie et du Muséum d’Histoire Naturelle de Genève 92(2): 421–460. 10.5962/bhl.part.81624 897542

[B29] Vít S (1990) Revision of the Neotropical species of the genus *Eucinetus* Germar (Coleoptera: Eucinetidae). Naturaliste Canadien 117(2): 103–122.

[B30] Vít S (1995) Deux espèces nouvelles d’Eucinetidae d’Amerique du Nord particulièrement intéressantes (Coleoptera: Eucinetidae). Elytron 9: 125–137.

[B31] Vít S (2000) Contribution à la connaissance de la famille Eucinetidae (Coleoptera). Revue Suisse de Zoologie 107: 123–138. 10.5962/bhl.part.80122 897542

[B32] Zhang SQ Che LH Li Y Liang D Pang H Ślipiński A Zhang P (2018) Evolutionary history of Coleoptera revealed by extensive sampling of genes and species. Nature Communications 9(1): 1–205. 10.1038/s41467-017-02644-4 PMC5768713 29335414

